# Therapeutic potentials of FexMoyS-PEG nanoparticles in colorectal cancer: a multimodal approach via ROS-ferroptosis-glycolysis regulation

**DOI:** 10.1186/s12951-024-02515-3

**Published:** 2024-05-16

**Authors:** Zhilong Yu, Chenyi Wang, Yingjiang Ye, Shan Wang, Kewei Jiang

**Affiliations:** 1 Department of Gastroenterological Surgery, Peking University People’s Hospital, Beijing, 100044 PR China; 2 Laboratory of Surgical Oncology, Beijing Key Laboratory of Colorectal Cancer Diagnosis and Treatment Research, Peking University People’s Hospital, Beijing, 100044 PR China

**Keywords:** Nanoparticles, Ferroptosis, Glycolysis, ROS, MAPK pathway

## Abstract

Improving cancer therapy by targeting the adverse tumor microenvironment (TME) rather than the cancer cells presents a novel and potentially effective strategy. In this study, we introduced Fe_x_Mo_y_S nanoparticles (NPs), which act as sequential bioreactors to manipulate the TME. Fe_x_Mo_y_S NPs were synthesized using thermal decomposition and modified with polyethylene glycol (PEG). Their morphology, chemical composition, and photothermal properties were characterized. The capability to produce ROS and deplete GSH was evaluated. Effects on CRC cells, including cell viability, apoptosis, and glycolysis, were tested through various in vitro assays. I*n vivo* efficacy was determined using CRC-bearing mouse models and patient-derived xenograft (PDX) models. The impact on the MAPK signaling pathway and tumor metabolism was also examined. The Fe_x_Mo_y_S NPs showed efficient catalytic activity, leading to increased ROS production and GSH depletion, inducing ferroptosis, and suppressing glycolysis in CRC cells. In vivo, the NPs significantly inhibited tumor growth, particularly when combined with NIR light therapy, indicating a synergistic effect of photothermal therapy and chemodynamic therapy. Biosafety assessments revealed no significant toxicity in treated mice. RNA sequencing suggested that the NPs impact metabolism and potentially immune processes within CRC cells. Fe_x_Mo_y_S NPs present a promising multifaceted approach for CRC treatment, effectively targeting tumor cells while maintaining biosafety. The nanoparticles exhibit potential for clinical translation, offering a new avenue for cancer therapy.

**Supplementary Information:**

The online version contains supplementary material available at 10.1186/s12951-024-02515-3.

## Introduction

Colorectal cancer (CRC) is a prevalent malignancy affecting the human digestive tract, representing a major global health challenge due to its high morbidity and mortality rates [1, 2]. The 2015 China Cancer Data report highlights the significant burden of CRC in China, with an estimated 383,000 new cases and 187,000 deaths annually. Among malignant tumors, CRC ranks third in incidence and fifth in mortality, posing a substantial threat to public health [3]. Traditional treatment options primarily consist of surgery, radiotherapy, and chemotherapy [4, 5]. However, these approaches often lead to challenges such as postoperative recurrence, metastasis, radiotherapy insensitivity, and chemotherapy resistance, which collectively diminish their clinical effectiveness [2, 6]. Consequently, recent research endeavors have placed a growing emphasis on identifying safe and efficacious treatments.

The tumor microenvironment (TME) is the complex milieu surrounding tumor cells, critical for tumorigenesis, growth, invasion, and metastasis [7–9]. Advances in nanotechnology and nanobiomaterials have revolutionized cancer diagnosis and targeted therapy, particularly within the TME [10, 11]. These innovations enable precise tumor-targeted diagnostics and treatments, and allow for the modulation of the TME using multifunctional nanoparticles (NPs) [12]. A new generation of intelligent nanoreactors, containing multivalent metal ions such as Fe^2+/3+^, Mo^4+/6+^, and Mn^2+/4+^, have shown exceptional catalytic activities. These nanoreactors are particularly beneficial for TME modulation, significantly improving the efficacy of various cancer treatments [13–15]. An example is the bimetallic oxide nanosheet FeWO_X_-PEG, which exhibits remarkable catalytic efficiency in catalyzing the decomposition of H_2_O_2_, generating O_2_ and hydroxyl radicals (•OH), and reducing endogenous GSH levels, thus enabling chemodynamic and immunological therapy [16]. Moreover, the adaptability of NPs for multifunctional modifications has spurred the design and utilization of diagnostic nanomedicines that offer simultaneous detection and treatment of cancer, marking significant strides in the field [17].

Reactive oxygen species (ROS) encompass various reactive oxygen-containing molecules, including free radicals and highly reactive non-radical intermediates produced during aerobic metabolism in living organisms [18]. Evidence suggests that the MAPK pathway is influenced by ROS, acting as a downstream conduit [19]. A wide array of proteins—ranging from cytokines and receptors to Ca^2+^ channel proteins, kinases/phosphatases, and transcription factors—plays critical roles in signal transduction and gene expression. These proteins are particularly sensitive to changes in the intracellular redox state [20–22]. ROS can modify the structure and function of these proteins by altering critical amino acid residues [23, 24]. In cancer therapy, the inhibition of cancer progression has been associated with increased ROS levels [25, 26]. Beck R et al. demonstrated apoptosis induction in K562 leukemia cells via the elevation of ROS, facilitated by ascorbic acid/menaquinone treatment. This process likely involved oxidative degradation of Hsp90, leading to the disruption of its chaperone function and subsequent degradation of both wild-type and mutant BCL-Abl proteins [27]. Similarly, You BR et al. reported that gallic acid treatment induced apoptosis in lung cancer A549 cells by potentially lowering mitochondrial membrane potential (MMP) and depleting glutathione (GSH), thus enhancing ROS production [28]. In nanomaterials, certain NPs containing low-valence metal ions catalyze Fenton or Fenton-like reactions with H_2_O_2_ in tumor-specific environments, generating significant quantities of ROS [29–31].

**Scheme 1:**
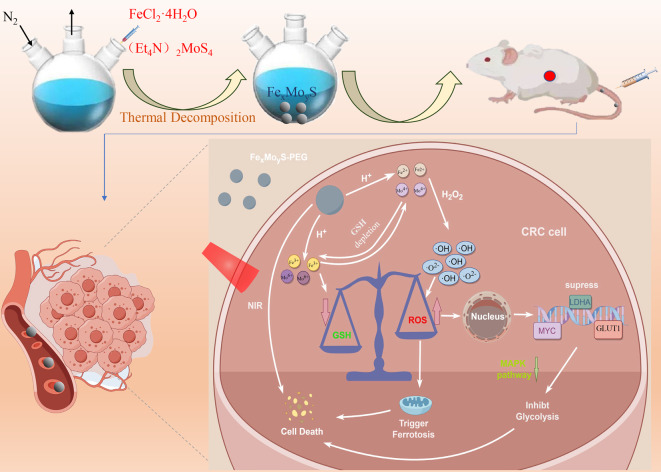
Scheme of the synthesis and therapeutic mechanism of Fe_x_Mo_y_S-PEG nanoparticle-based ROS-ferroptosis-glycolysis regulation in CRC

This study introduced the innovative development of Fe_x_Mo_y_S NPs, featuring bimetallic sulfides and containing multivalent metal elements. These NPs acted as intelligent cascade bioreactors, effectively altering the TME and enhancing multimodal treatment strategies for CRC. The Fe_x_Mo_y_S-PEG NPs, modified with poly(ethylene glycol) (PEG), showcased an impressive ability to generate •OH via the catalytic decomposition of H_2_O_2_, facilitating •OH-induced chemo-dynamic therapy (CDT). Furthermore, these NPs reduced endogenous GSH levels, amplifying oxidative stress within tumors and thereby enhancing CDT efficacy. The synergistic effect of GSH depletion and •OH production in these bioreactors was bolstered by the cyclic redox reactions between Fe^2+^/Mo^4+^ and Fe^3+^/Mo^6+^. Experimental results revealed that Fe_x_Mo_y_S-PEG NPs excelled in producing •OH and depleting GSH in cancer cells. In vitro, these NPs suppress the growth, induce apoptosis, and inhibit the metastasis of CRC cells. Additionally, in patient-derived xenograft (PDX) models, Fe_x_Mo_y_S-PEG effectively hindered tumor proliferation. RNA sequencing analysis highlighted that Fe_x_Mo_y_S-PEG bioreactors induced significant ROS production, which was instrumental in promoting ferroptosis and inhibiting glycolysis. In summary, the findings demonstrated that these innovative bioreactors triggered ferroptosis and suppressed glycolysis, thereby impeding the growth and dissemination of CRC cells through modulation of the MAPK signaling pathway (Scheme 1).

## Materials and methods

### Synthesis of Fe_x_Mo_y_S-PEG NPs

Fe_x_Mo_y_S NPs were synthesized via a conventional organic-phase method [16]. In a typical procedure, a mixture containing FeCl_2_·4H_2_O (20 mg), (Et_4_N)_2_MoS_4_ (40 mg), and oleylamine (25 ml) was combined in a three-necked flask and subjected to vigorous magnetic stirring. This mixture was then heated to 95 °C under a nitrogen atmosphere and maintained at this temperature for 8 h. Subsequently, the temperature of the solution was increased to 260 °C and maintained for 30 min. After cooling naturally to room temperature, a black oily mixture was obtained. To isolate the NPs, chloroform (5 mL) and ethanol (60 mL) were added to the mixture, followed by dispersion centrifugation to yield a black precipitate. This was then washed several times with chloroform and anhydrous ethanol and dried under vacuum at room temperature for 4 h to produce a black powder. For surface modification, the hydrophobic Fe_x_Mo_y_S NPs were treated with DSPE-PEG. Specifically, 10 mg of Fe_x_Mo_y_S NPs were mixed with 5 mg of DSPE-PEG in 4 mL of chloroform, and the mixture was subjected to stir for 12–24 h and centrifuged. The resultant solution was washed three times with ethanol and deionized water, then dried using a rotary evaporator. The final Fe_x_Mo_y_S-PEG sample was reconstituted in deionized water and stored at 4 °C for subsequent use.

### Characterization of Fe_x_Mo_y_S-PEG

Transmission electron microscopy (TEM) was employed to examine the morphological characteristics of the NPs. The chemical compositions and specific elemental configurations were determined using X-ray photoelectron spectroscopy (XPS). Determination of absorbance of NPs by UV-VIS absorption spectrometry. Subsequently, different concentrations of FexMoyS-PEG NPs (100, 200, and 400 µg/ml) were subjected to an 808 nm near-infrared (NIR) laser in an aqueous environment. The photothermal conversion efficiency (η) was evaluated by recording the temperature changes of the solution during the heating and cooling processes, utilizing a thermal imaging camera for real-time monitoring [32].

### Assessing the ability of the NPs to generate ROS

Samples of Fe_x_Mo_y_S-PEG (1.5 mL at concentrations of 0–200 µg/mL) were mixed with H_2_O_2_ (900 µL at 100 mM) and DPBF (100 µL at 1 mg/mL) in (PBS (pH 6.5, 3 mL). The formation of ROS in the solution was monitored by recording the UV–Vis absorption spectra, specifically noting the changes in absorbance at 410 nm. This method allows for the quantitative analysis of ROS generated in the reaction mixture.

### Depletion of GSH

The depletion of GSH by Fe_x_Mo_y_S-PEG NPs was evaluated using Ellman’s assay. In short, various concentrations of Fe_x_Mo_y_S-PEG (0, 200, 400, and 800 µg/mL) were mixed with 2 mM GSH in a shaker for adequate incubation. As a part of the experimental setup, centrifuge tubes containing a mixture of 200 mM H_2_O_2_ and 2 mM GSH served as positive controls, indicating expected reactions, while tubes with only 2 mM GSH acted as negative controls, representing baseline conditions. Following centrifugation, 10 µL of DTNB was introduced to the supernatant. The subsequent reaction between DTNB and any remaining GSH resulted in the formation of 5-thio-2-nitrobenzoic acid, which was quantitatively assessed by measuring the absorbance at 410 nm using UV-Vis absorption spectroscopy.

### Cellular experiments

HCT116 and NCM460 cells were seeded in a 96-well plate (10,000 cells per well) and incubated for 24 h at 37 °C with 5% CO_2_. Subsequently, the cells were treated with different concentrations of Fe_x_Mo_y_S-PEG for an additional 24 h, followed by a further incubation period of 18 h. Cell viability was assessed using a Cell Counting Kit-8 (CCK-8) (Yeasen, China). HCT116 and NCM460 cells were purchased from Type Culture Collection of the Chinese Academy of Science.

In the CCK-8 assay, HCT116 cells were plated at a density of 3 × 10^3^ cells per well in 96-well plates. Post-treatment, 10 µL of CCK-8 solution was added to each well, and the cells were incubated for 2 h in the dark. Absorbance at 450 nm was measured every 24 h using a microplate reader (BioTek Instruments, USA). Cell proliferation was evaluated using an EdU cell proliferation assay (Beyotime, China). Treated HCT116 cells were seeded at a density of 2 × 10^4^ cells/mL in a volume of 100 µL in 96-well plates. Cells were incubated with 50 mM EdU for 2 h at 37 °C. Following fixation with 4% paraformaldehyde and permeabilization with 1% Triton X-100, cells underwent staining with the Click reaction cocktail and Hoechst 33,342 for visualization. The stained cells were analyzed both visually and quantitatively using a fluorescence microscope.

In this experimental setup, 40,000 cells were seeded into the upper chamber of a transwell plate (Corning, USA) featuring 8.0 μm pores, using 400 µL of serum-free medium. Two experimental conditions were established: one using Matrigel (BD, USA) to simulate invasion and another without Matrigel for assessing cell migration. The lower compartment contained 600 µL of DMEM supplemented with 10% FBS. Following a 24-hour incubation, cells in the upper chamber were fixed with 4% formaldehyde for 30 min and subsequently stained with 0.1% crystal violet for another 30 min to facilitate cell counting.

For the in vitro ROS staining, HCT116 cells were seeded in confocal dishes and incubated for 24 h. They were then treated with Fe_x_Mo_y_S-PEG (200 µg mL^− 1^) and H_2_O_2_ (100 μm) for 8 h. Post-treatment, cells were stained with DCFH-DA (20 µM) for 30 min to detect ROS generation.

Cell viability was assessed using a dual-staining approach with calcein AM (for live cells) and propidium iodide (PI, for dead cells). HCT116 cells were cultured in six-well plates overnight, treated with NPs (200 µg/mL) and/or H_2_O_2_ (100 mM) for 6 h, washed, and then stained with calcein AM and PI solutions for 15 min before fluorescence imaging.

Finally, apoptosis was evaluated using an Annexin V/PI Apoptosis Detection Kit (Yeasen, China). Post-treatment, cells were resuspended in a binding buffer containing Annexin V and PI and incubated in the dark for 15 min. The extent of apoptosis was quantitatively analyzed using a FACS Celesta flow cytometer.

### Measuring intracellular metabolites and the extracellular acidification rate (ECAR)

The ECAR was determined following the XF Glycolysis Stress Test protocol using a Seahorse XF96 Extracellular Flux Analyzer (Agilent Technologies, USA). Glucose utilization was quantified using glucose assay kits (Beyotime, China), while lactate production was assessed via lactate content assay kits (Sangon, China), adhering strictly to the instructions provided with each kit.

### RNA sequencing

Microarray analysis was conducted at the Laboratory of the Major Biotechnology Company (Shanghai, China). HCT116 cells were prepared and divided into two groups: the normal control (NC) and the treatment group (200 µg mL^− 1^ + H_2_O_2_). Following a further 6-hour incubation, total RNA was extracted using the mirVana miRNA Isolation Kit (Ambion). RNA quality was assessed using an Agilent 2100 Bioanalyzer (Agilent Technologies, Santa Clara, CA, USA), with only samples having an RNA integrity number (RIN) of 7 or above being selected for further analysis. Library construction was performed using the TruSeq Stranded mRNA LT Sample Prep Kit (Illumina, USA). Sequencing was carried out on an Illumina HiSeq™ 2500 platform, generating paired-end reads of 125–150 bp in length.

### PCR and Western blot

Total RNA was extracted from various HCT116 cell samples using TRIzol reagent (TaKaRa, Shiga, Japan). The extracted RNA was then reverse-transcribed to complementary DNA (cDNA) utilizing a Strand cDNA Synthesis Kit (Yeasen, China). Quantitative real-time polymerase chain reaction (qRT-PCR) was performed to measure RNA expression, employing SYBR Green Master Mix (Yeasen, China) on an ABI 7900HT System (Applied Biosystems, USA), with GAPDH serving as the internal control for mRNA quantification. Expression levels were determined using the ΔΔCt method [33]. The primer sequences are detailed in Table S1.

For protein analysis, samples were processed through electrophoresis on 10% SDS-PAGE gels and then transferred to PVDF membranes (Millipore). Membranes were blocked in 5% skim milk for 2 h before incubation with primary antibodies. This was followed by incubation with secondary antibodies at a 1:1000 dilution for one hour. After three washing steps, proteins were detected using an enhanced chemiluminescence (ECL) reagent (Millipore, MA, USA). β-actin or GAPDH served as the loading controls to ensure accuracy in protein quantification.

### Animal experiments

The animal experiments were conducted in strict accordance with the “China National Standards for the Care and Use of Laboratory Animals” and were approved by the Ethics Committee of Peking University People’s Hospital. The xenograft mouse models were randomly divided into four groups, each comprising five mice. The first and second groups received intravenous infusions of PBS (200 µL), while the third and fourth groups were treated with NPs at a dosage of 2 mg/mL (200 µL). Eight hours post-administration, groups two and four were exposed to an 808 nm laser (1.0 W/cm²) for ten minutes. The thermal effects were monitored using a thermal imaging camera. The progression of the treatment was evaluated by measuring the tumors’ sizes (calculated as 0.56 × length × width²) and the mice’s weights every two days. After 18 days of treatment, vital organs were harvested for preservation in formalin, followed by detailed examination through hematoxylin and eosin (H&E) staining and immunohistochemical (IHC) analysis.

The PDX model of CRC was developed by transplanting fresh tumor tissues from two CRC patients into NSG mice. When the tumors reached 150 mm³, they were segmented and subcutaneously engrafted into 4-5-week-old male NSD mice. Once the tumors grew to about 100 mm³, the mice were randomly separated into two groups: a PBS group and an NP group, with each containing five mice. Treatments were administered via intravenous injections in the tail vein. Tumor sizes were monitored biweekly throughout the experiment. Following around four weeks of treatment, the mice were humanely euthanized, and the tumors were excised and weighed. The collected tumor tissues were then processed for histological evaluation through H&E staining and IHC analysis. This study received the necessary approval from the Ethics Committee of Peking University People’s Hospital.

### Statistics

Statistical analyses were conducted using GraphPad Prism Software (GraphPad Software, USA). Data are presented as means ± standard deviations (SDs). Analysis of variance (ANOVA) was applied for comparisons involving more than two groups. Significance levels were denoted as follows: *p < 0.05, **p < 0.01, and ***p < 0.001.

## Results and discussion

### Characteristics of the Fe_x_Mo_y_S-PEG NPs

**Fig. 1:**
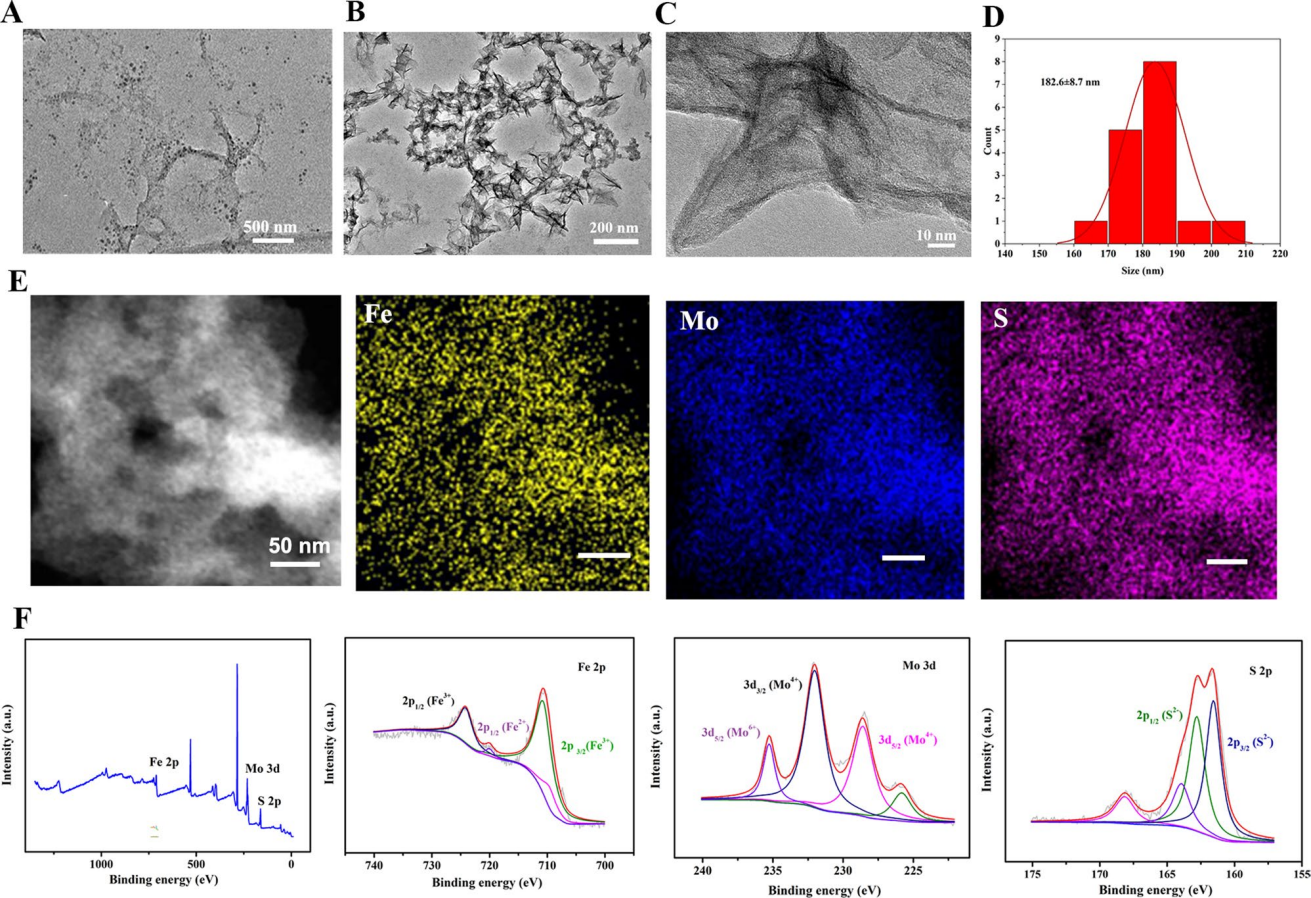
Characterization of Fe_x_Mo_y_S-PEG NPs. Low- (**A**), high- (**B**), and (**C**) HR-TEM images of NPs. (**D**) Hydrodynamic diameter distribution diagram of NPs. (**E**) Element mapping images of Fe, Mo, and S in NPs. (**F**) XPS spectra of survey and Fe 2p, Mo 3d, and S 2p of the NPs. Scale bar, 50 nm

In this study, Fe_x_Mo_y_S-PEG NPs were synthesized using a thermal decomposition method in the organic phase. The morphology of the NPs, as observed through TEM, displayed uniform Fe_x_Mo_y_S-PEG NPs (Fig. 1A and B). High-resolution TEM further identified the lattice spacing of the NPs to be approximately 0.197 nm, indicative of their crystalline structure (Fig. 1C). Based on TEM micrographs, the average size is 182.6 ± 8.7 nm which is considerably monodisperse for synthesized NPs (Fig. 1D). Elemental mapping confirmed the homogeneous distribution of Fe, Mo, and S within the NPs (Fig. 1E), supporting the composition specified for the synthesized NPs. XPS was utilized to analyze the chemical state of the NPs, revealing distinct peaks that correspond to Fe 2p, S 2p, and Mo 3d, affirming the presence of these elements in their anticipated chemical states (Fig. 1F). Additionally, the dynamic hydrated diameter of the NPs, determined via Zetasizer analysis, was found to be around 203 nm, as shown in Fig. S1. Specifically, the XPS spectra indicated the presence of Fe 2p peaked at 724.0 eV, 721 eV, and 713 eV, representing the 2p_1/2_ and 2p_3/2_ states, respectively. Additionally, Mo^4+^ 3d_5/2_ and 3d_3/2_ showed binding energies of 228.5 eV and 232.01 eV, respectively, while Mo^6+^ had a binding energy of 235.5 eV. The identification of Fe^2+^ and Mo^4+^ was particularly notable due to their potential roles in catalyzing the conversion of H_2_O_2_ into •OH, thus enhancing the efficacy of CDT. The presence of Fe^3+^ and Mo^6+^ peaks suggests conditions favorable for O_2_ generation (Fig. 1F), which could be beneficial for cancer treatment strategies. The S 2p spectrum showed peaks at 164.0 eV and 164.95 eV corresponding to the S^2−^ state, completing the characterization of the NPs. These collective findings corroborate the successful synthesis of the Fe_x_Mo_y_S-PEG NPs with the intended chemical composition and structural properties, laying a foundation for their potential application in cancer therapy.

### Photothermal property of Fe_x_Mo_y_S-PEG NPs

**Fig. 2:**
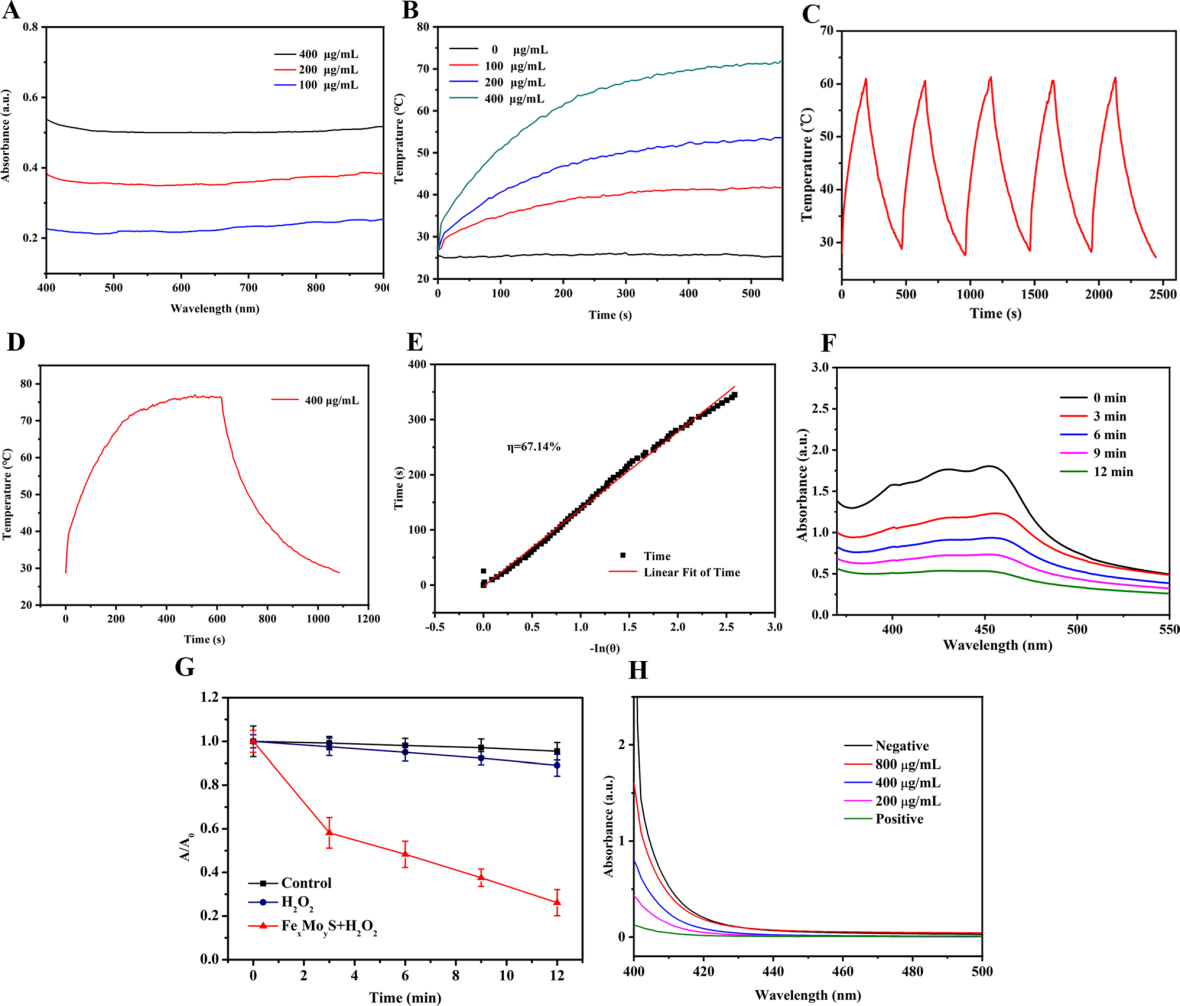
Photothermal and chemodynamic effects of NPs. (**A**) UV‒Vis absorption spectra of the NPs. (**B**) Temperature increase with various concentrations (0, 100, 200, 400 µg/mL) of NPs. (**C**) The photothermal stability of NPs (200 µg·mL-1) under laser irradiation (808 nm) for 5 cycles. (**D**) Temperature curve of rising with irradiation and naturally cooling. (**E**) Linear regression curve (red) of the cooling process. (**F**) UV‒Vis absorption of DPBF at 410 nm for the control group and NP group. (**G**) Different treatments result in the time-dependent degradation of methylene blue, indicating ·OH production. (**H**) Different concentrations of NPs induced the depletion of GSH

In this study, an 808 nm near-infrared laser with a power density of 1 W/cm² was employed to irradiate the NP solution. Temperature changes during this process were documented using an infrared thermal imager. The NPs demonstrated strong absorption across the visible to near-infrared range (Fig. 2A), suggesting their efficiency in photothermal conversion. Specifically, a significant increase in the solution’s temperature to 47 °C was observed following 10 min of continuous laser exposure (400 µg/mL concentration) (Fig. 2B). To assess the photostability of the Fe_x_Mo_y_S-PEG NPs, we conducted five successive on/off laser cycles, observing a negligible decrease in temperature, indicative of the NPs’ robust photostability (Fig. 2C). The photothermal conversion efficiency (η) of the NPs was determined by analyzing the temperature variations during the heating and cooling phases (Fig. 2D and E). Remarkably, the efficiency value, η, was calculated to be 67.14%, which surpasses the efficiencies reported for traditional photothermal therapy (PTT) [34–36]. These results underscore the superior photothermal capabilities of Fe_x_Mo_y_S-PEG NPs, establishing their promise as effective agents in PTT applications.

### ROS and GSH detection in vitro

The Fe_x_Mo_y_S-PEG NPs contain Fe^2+^/Fe^3+^ and Mo^5+^/Mo^6+^ redox pairs, providing a significant advantage in modifying the TME by elevating ROS levels and diminishing GSH concentrations. The capacity of Fe_x_Mo_y_S-PEG NPs to generate ROS, specifically [1]O2 and •OH, was evaluated using the chemical probe DPBF under US-vis irradiation. A progressive decline in DPBF absorption intensity over time was observed, signifying ROS production by the NPs as a result of DPBF degradation under US-vis illumination (Fig. 2F). The reduction in DPBF absorption, particularly at 410 nm, was attributed to its irreversible reaction with ROS via the 1,4-cycloaddition process. Among different experimental setups, the combination of NPs with H_2_O_2_ exhibited the most significant DPBF degradation, indicating the highest level of ROS generation (Fig. 2G). These results underscore the role of Fe and Mo ions within the NPs in facilitating ROS production upon laser activation, pointing to their potential effectiveness in enhancing tumor-targeted therapies through oxidative stress modulation.

GSH serves as a principal antioxidant, crucial for maintaining the redox balance within organisms [37]. There is a growing consensus that lowering GSH levels could enhance the efficacy of dynamic therapeutic modalities, such as radiotherapy [38–40]. In this context, the ability of Fe_x_Mo_y_S-PEG NPs to reduce GSH levels was evaluated using a GSH assay kit. Following the addition of DTNB to the NP solution, GSH concentrations were recorded at several time intervals, revealing a significant decrease over time. This observation underscores the potent capability of Fe_x_Mo_y_S-PEG to catalyze the generation of •OH and reduce GSH levels (Fig. 2H). Such results highlight the potential efficacy of NPs with multivalent metal elements in eradicating cancer cells, especially in the presence of endogenous H_2_O_2_.

### Fe_x_Mo_y_S-PEG NPs inhibited growth and metastasis in vitro

**Fig. 3:**
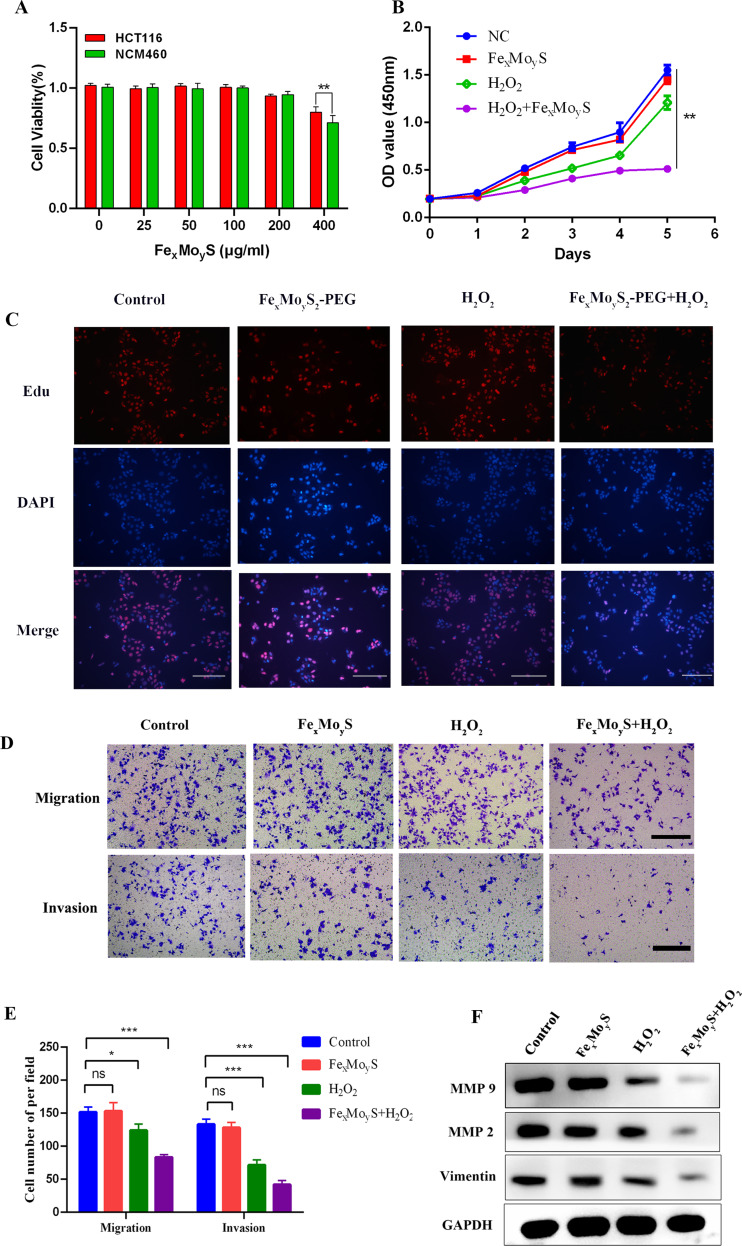
Fe_x_Mo_y_S-PEG inhibited the proliferation and metastasis of HCT116 cells. (**A**) The viability of HCT116 and NCM460 cells was assessed after coculture with NPs at various concentrations. (**B**) Cell viability CCK-8 assay in different groups. (**C**) EdU incorporation assay for cell proliferation. Scale bar, 250 μm. (**D-E**) Transwell migration and invasion assays were performed on HCT116 cells in various groups. Scale bars, 100 μm. (**F**) Western blot analysis was performed to assess the protein levels of MMP9, MMP2, and Vimentin in the different treatment groups. * *p* < 0.05, ** *p* < 0.01, ***p < 0.001

The biocompatibility of NPs is essential for their application in biological contexts [41]. In this research, the cytotoxicity of NPs was evaluated using standard CCK-8 assays on HCT116 and NCM460 cells. The results indicated excellent biocompatibility of the NPs (Fig. 3A). However, at higher concentrations (400 µg/ml), the NPs slightly increased cytotoxic effects in cancer cells, likely due to a chemodynamic reaction. To mimic the TME, H_2_O_2_ was added during cell treatment, dividing the cancer cells into four groups for comparison: Control, H_2_O_2_, NP, and NPs + H_2_O_2_. The CCK8 assays demonstrated that the combination of NPs and H_2_O_2_ significantly inhibited the proliferation of HCT116 cells (Fig. 3B). This inhibitory effect was further confirmed by the EdU incorporation assay, where a substantial reduction in EdU-positive cells was observed in the NP + H_2_O_2_ group, suggesting reduced cell proliferation (Fig. 3C). The capability of the cancer cells to migrate and invade was also assessed through Transwell migration and invasion assays, with the NP + H_2_O_2_ group showing a marked decrease in both migration and invasion abilities (Fig. 3D and E). Additionally, Western blot analysis further corroborated these findings, indicating that the combined NP + H_2_O_2_ treatment significantly reduced the expression of metastasis-related proteins such as MMP-9, MMP-2, and Vimentin (Fig. 3F). MMP-2, MMP-9 and Vimentin are key proteins that affect the process of EMT, which are highly expressed in various tumor tissues and play an important role in tumor development and metastasis. These in vitro experiments support the conclusion that Fe_x_Mo_y_S-PEG NPs, particularly when combined with H_2_O_2_, possess tumor-suppressive capabilities in colorectal cancer cells.

### **Fe**_**x**_**Mo**_**y**_**S-PEG NPs promoted CRC cell death and apoptosis**

**Fig. 4:**
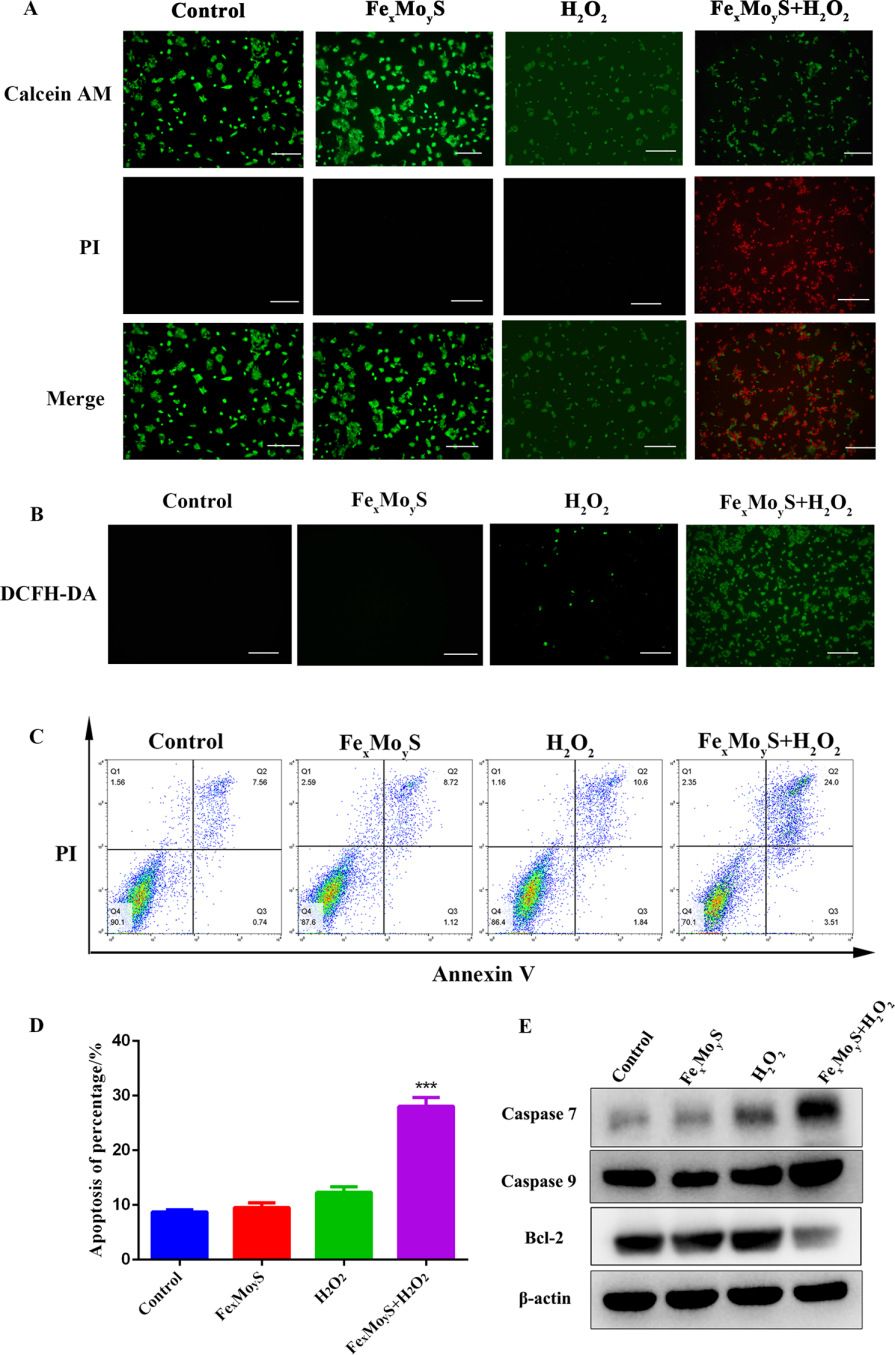
Fe_x_Mo_y_S-PEG can produce a large amount of ROS and promote cell death and apoptosis in colorectal cancer cells. (**A**) Fluorescence images depicting the viability of cells following various treatments. (Control, H_2_O_2_, NPs and NPs + H_2_O_2_). (**B**) Fluorescence images of intracellular ROS production in HCT116 cells in various groups. (**C**) Flow cytometric detection of apoptosis in the different groups. (**D**) Flow cytometric histograms of apoptosis detection. (**E**) Western blot analysis was performed to assess the protein levels of Casepase 7, Casepase 9 and Bcl-2 in the different treatment groups. Scale bar, 250 μm. ***p < 0.001

In the study, cell viability following NP treatment was evaluated using calcein-AM and PI staining. Viable cells were marked by green fluorescence, while dead or dying cells exhibited red fluorescence. Compared to the control group, a significant increase in apoptosis was observed in the cells treated with NPs and H_2_O_2_ (Fig. 4A). The production of ROS was investigated using confocal microscopy and DCFH-DA staining. An increase in ROS generation was indicated by intensified green fluorescence in HCT116 cells treated with both NPs and H_2_O_2_, highlighting the role of the NPs in facilitating ROS production (Fig. 4B). To delve deeper into the NPs’ biological impact, apoptosis in HCT116 cells was assessed using Annexin V/PI staining. The results, represented in Fig. 4C and D, showed that treatment with NPs and H_2_O_2_ led to a significant increase in apoptotic cells. Additionally, treatment with NPs and H_2_O_2_ resulted in an elevation of Caspase 7 and Caspase 9 levels and a marked reduction in BCL-2 levels (Fig. 4E). These changes corroborate the apoptosis observations made via flow cytometry. These results collectively suggest that Fe_x_Mo_y_S-PEG NPs, particularly in conjunction with H_2_O_2_, possess considerable potential for CDT applications in CRC management, highlighting their ability to induce cell death and enhance ROS production.

### RNA sequencing

**Fig. 5:**
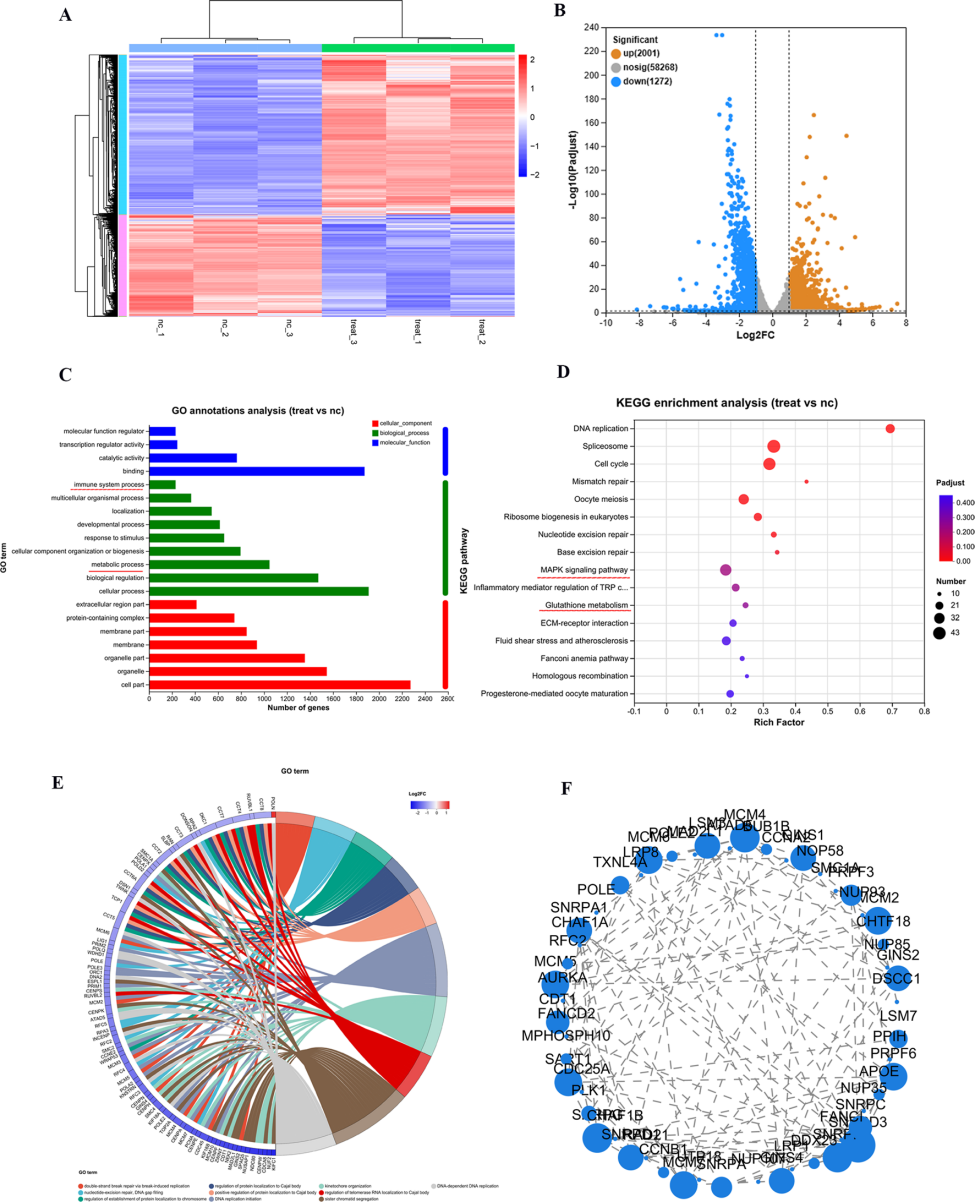
RNA sequencing of HCT116 cells following various treatments. (**A**) Cluster diagram of DEGs between the NP-treated (Fe_x_Mo_y_S-PEG + H_2_O_2_) group and the NC (control) group. (**B**) Volcano plots comparing the downregulated and upregulated genes in the NP-treated groups with those in the control groups. (**C**) GO annotation analysis of DEGs. (**D**) The results of KEGG pathway enrichment analysis (top 200 pathways). (**E**) A circus heatmap of metabolism-related DEGs correlated with the NP-treated group. (**F**) Network of interactions between these essential genes

To elucidate the therapeutic effects of Fe_x_Mo_y_S-PEG NPs on CRC cells, an extensive analysis was conducted on HCT116 cell samples. The reliability of RNA-sequencing data was confirmed through unsupervised hierarchical clustering, which showed significant grouping among samples from identical treatment groups (Fig. S2. This analysis identified 5,364 differentially expressed genes (DEGs) between the NPs + H_2_O_2_ group and the control group, with 2,001 genes upregulated and 1,272 genes downregulated (Fig. 5A and B). Gene Ontology (GO) annotation analysis was performed to categorize these DEGs, revealing that genes affected by Fe_x_Mo_y_S-PEG NPs treatment were significantly associated with immune system processes and metabolic functions (Fig. 5C). Furthermore, Kyoto Encyclopedia of Genes and Genomes (KEGG) pathway analysis indicated that these DEGs were predominantly enriched in the MAPK signaling pathway and pathways involved in glutathione metabolism, emphasizing the link between nanoparticle treatment and metabolic alterations triggered by ROS (Fig. 5D). GO enrichment analysis further supported these findings, highlighting the significant role Fe_x_Mo_y_S-PEG NPs play in modulating metabolic activities in the treatment of CRC (Fig. 5E). Additionally, the analysis of the protein-protein interaction network among the DEGs revealed key proteins implicated in tumor progression (Fig. 5F). These findings suggest a strong interconnection between altered metabolism and cell death processes induced by Fe_x_Mo_y_S-PEG NPs in CRC cells, indicating the potential mechanisms through which these NPs exert their therapeutic effects.

### Fe_x_Mo_y_S-PEG NPs promoted ferroptosis and inhibited glycolysis in HCT116 cells

**Fig. 6:**
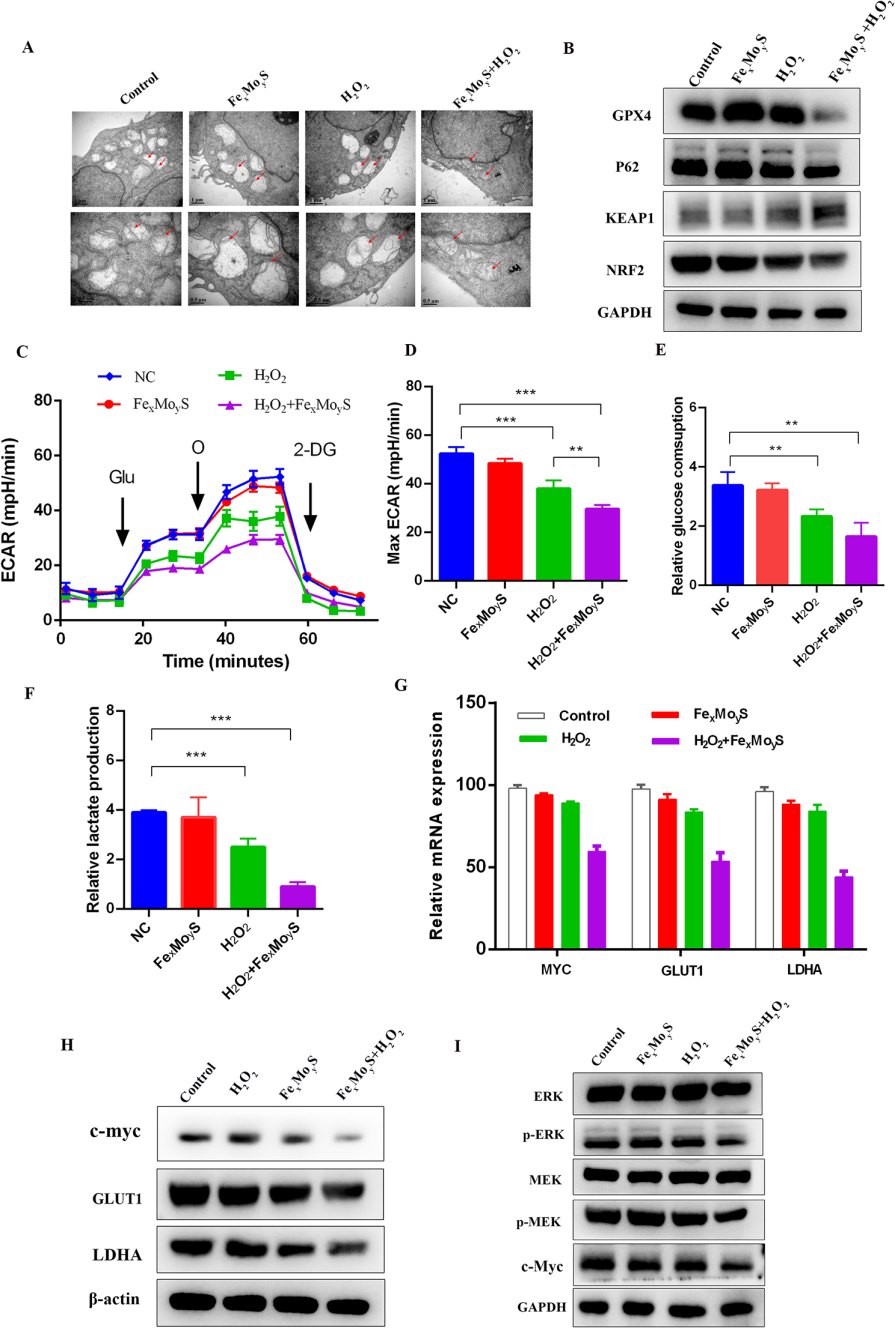
Fe_x_Mo_y_S-PEG promoted ferroptosis and inhibited glycolysis in HCT116 cells. (**A**) Representative characteristics of ferroptosis were examined via transmission electron microscopy. (**B**) Western blot analysis was also conducted to evaluate the levels of proteins associated with ferroptosis in the different experimental groups. (**C**) The ECAR was measured in HCT116 cells subjected to various treatments using an XF Extracellular Flux Analyser. Glucose, oligomycin, and 2-DG were injected sequentially at different time intervals. The presented data depict the results of three separate experiments. (**D**) The maximum ECAR reflects the glycolytic capacity of the different treatment groups. (E) Relative glucose consumption and (**F**) lactate production in HCT116 cells in the various groups. (**G**) The mRNA levels of MYC, GLUT-1 and LDHA in the different treatment groups were measured by qRT‒PCR. (**H**) The MYC, GLUT-1 and LDHA protein levels in the different groups were determined by western blot analysis. (**I**) ERK, MEK, p-MEK, and p-ERK were detected by western blot after the different treatments. ** *p* < 0.01, ***p < 0.001

In this study, HCT116 cells treated with Fe_x_Mo_y_S-PEG NPs combined with H_2_O_2_ demonstrated a significant increase in ROS production. It is well-documented that ROS influences the metabolic phenotype of cancer cells [42]. KEGG enrichment analysis revealed a strong association between glutathione metabolism and the induction of ferroptosis in cancer cells [43]. Further investigation into the effects of NPs on ferroptosis in vitro revealed notable changes in cell morphology through electron microscopy. Specifically, cells in the NPs + H_2_O_2_ group showed considerable mitochondrial shrinkage and loss or reduction of mitochondrial cristae (Fig. 6A), which are characteristic signs of ferroptosis. Additionally, there was a noticeable alteration in the expression of proteins related to ferroptosis in treated cells. Levels of GPX4, a key enzyme that protects against lipid peroxidation and ferroptosis, were found to decrease. Similarly, reductions in P62 and NRF2 protein levels were observed, alongside an increase in KEAP1 expression following treatment with NPs and H_2_O_2_ (Fig. 6B). These protein expression changes support the hypothesis that Fe_x_Mo_y_S-PEG NPs facilitate the induction of ferroptosis in cancer cells.

Glycolysis is widely recognized as the primary energy source for tumor cells, supporting their proliferation and growth [44, 45]. In this study, RNA sequencing highlighted significant changes in glycolysis-related genes, such as LDHA and HK2, in HCT116 cells treated with Fe_x_Mo_y_S-PEG NPs combined with H_2_O_2_. This suggests that the NPs may inhibit tumor cell proliferation by suppressing glycolytic activity. Further investigations were conducted using qPCR to examine changes in the expression of key glycolysis-related genes, including MYC, LDHA, and GLUT1, in cells exposed to various treatments. The results showed significant downregulation of these genes in the NPs + H_2_O_2_ group, indicating a suppression of glycolytic processes in tumor cells (Fig. 6C). To provide additional confirmation, we measured the extracellular acidification rate, glucose consumption, and lactate production, which are indicative of glycolytic activity. The results revealed a notable reduction in these parameters in the NPs + H_2_O_2_ group, underscoring the NPs’ impact on reducing glycolytic capacity in CRC cells (Fig. 6D-F). Western blot analysis supported these findings by showing downregulation of MYC, LDHA, and GLUT1 proteins, consistent with the qPCR results (Fig. 6G, H). The potential role of the MAPK signaling pathway in the mechanism of action of the NPs was also explored, given its known association with CRC development [46]. Examination of specific proteins in the MAPK pathway revealed that the NPs + H_2_O_2_ treatment led to reduced phosphorylation of MEK and ERK, indicating suppression of the MAPK pathway (Fig. 6I). Since MYC, a key regulator of glycolysis, is influenced by the MAPK pathway and can affect glycolytic genes [47, 48], our results suggested that the NPs disrupted CRC cell glycolysis via this pathway, contributing to tumor growth inhibition. Collectively, Fe_x_Mo_y_S-PEG NPs exert a therapeutic effect on CRC cells by impacting metabolic pathways, specifically through the suppression of glycolysis and modulation of the MAPK signaling pathway, leading to reduced tumor cell proliferation and energy metabolism.

### In vivo antitumor efficacy of Fe_x_Mo_y_S-PEG NPs combined with NIR light

**Fig. 7:**
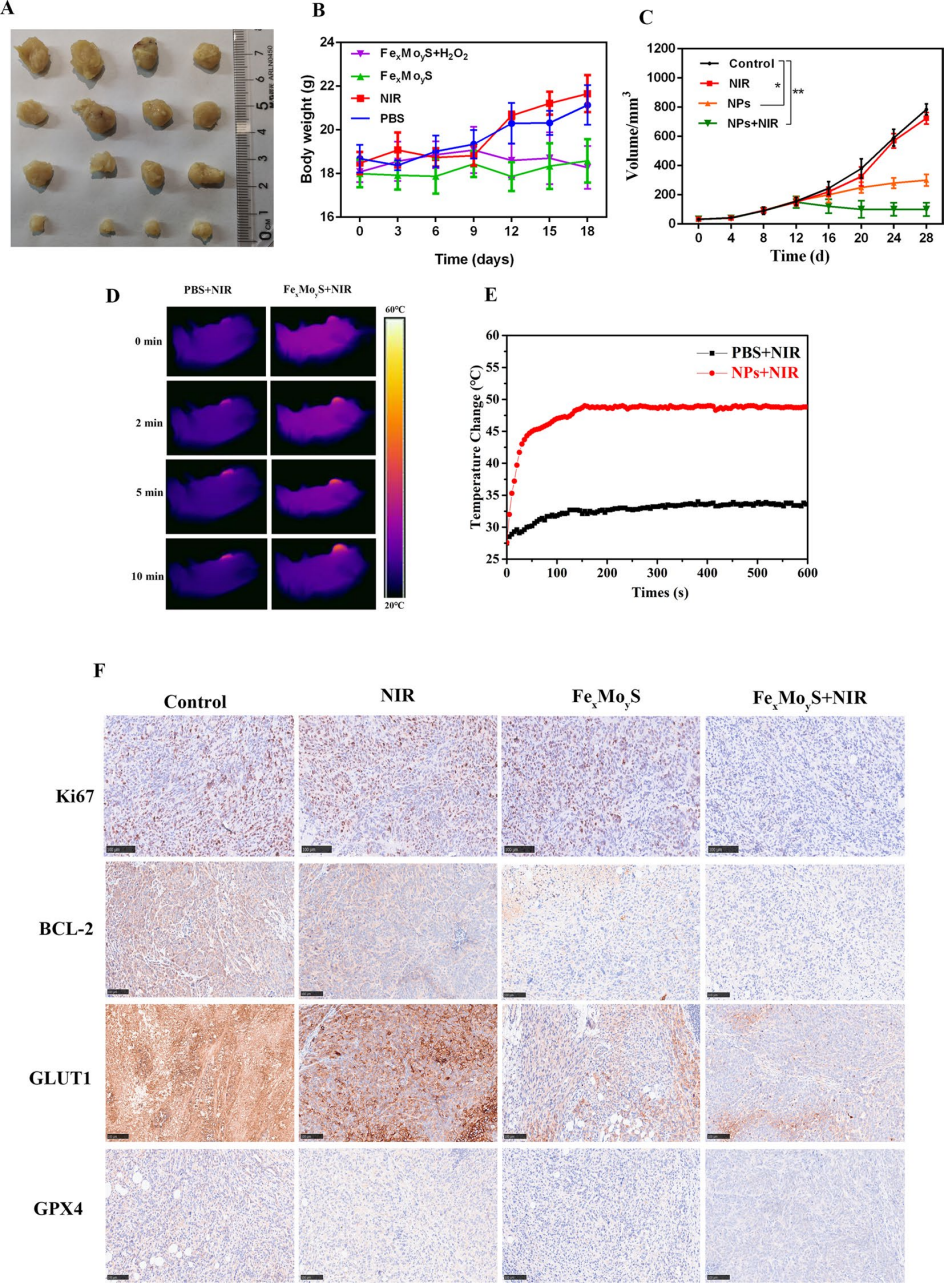
Antitumour activity of Fe_x_Mo_y_S-PEG in nude mouse tumor cell xenografts. (**A**) Representative xenograft images of HCT116 tumors in the subcutaneous area. (**B**) Changes in the weights of tumor-bearing nude mice in the different groups. (**C**) Changes in tumor volume among the different groups. (**D**) Temperature changes and (**E**) infrared thermographic imaging of mice injected with PBS or NPs during laser exposure. (**F**) Ki67, BCL-2, GLUT1 and GPX4 immunostaining of the tumors in the control group and the remaining treated cohorts. (scale bar, 100 μm). * *p* < 0.05, ** *p* < 0.01

To explore the impact of Fe_x_Mo_y_S-PEG NPs on in vivo cellular proliferation, a xenograft colorectal cancer model was developed in nude mice. Tumor volume measurements conducted biweekly revealed that NP application notably decelerated tumor growth compared to the control, as evidenced in Fig. 7A-C. Notably, tumors subjected only to NIR light showed growth rates comparable to the control group, underscoring the inefficacy of NIR light alone in halting tumor expansion. Conversely, the application of both NPs and NIR light significantly hindered tumor growth, suggesting a synergistic effect of PTT and CDT. In procedures involving NIR light, an 808 nm laser was utilized to irradiate the mice, simultaneously monitoring thermal signals and recording temperatures in the tumor vicinity. Upon laser exposure, temperatures within the NP-treated tumors rose rapidly to 50 °C, a level known from previous studies to induce cancer cell death through apoptosis and necrosis when maintained above 42 °C. In contrast, tumors in the control group exhibited only minor temperature increases, not exceeding 35 °C (Fig. 7D and E). Further, IHC staining of xenograft tumor tissues, targeting markers such as GPX4, Glut1, Bcl2, and Ki-67, revealed that NP + NIR treatment significantly reduced the expression of these proteins (Fig. 7F). These findings, aligning with laboratory observations, confirm the efficacy of Fe_x_Mo_y_S-PEG NPs in inhibiting the progression of CRC in vivo, showcasing their therapeutic potential.

### Biosecurity of Fe_x_Mo_y_S-PEG NPs

**Fig. 8:**
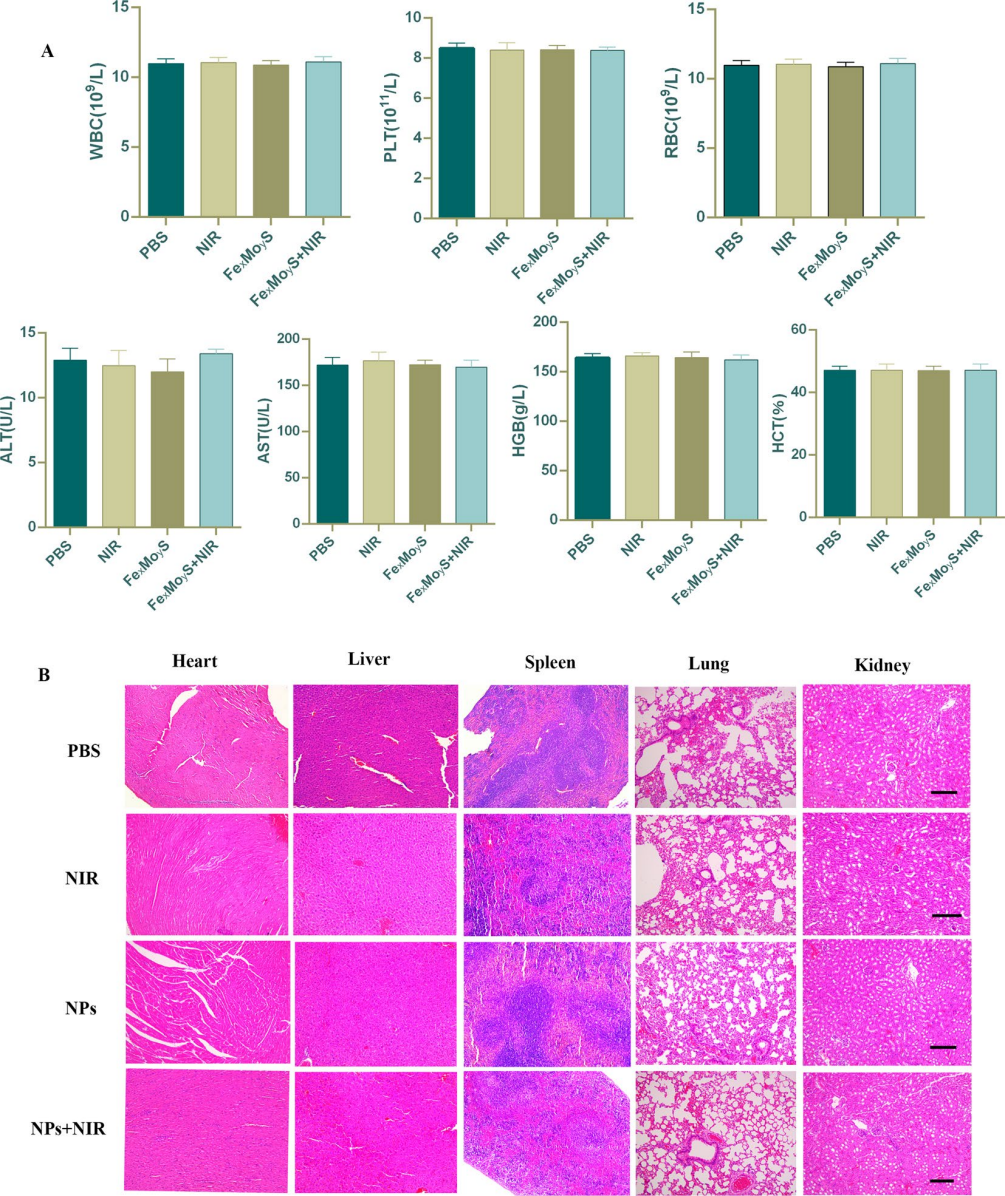
Biosafety of Fe_x_Mo_y_S-PEG in nude mice. (**A**) Blood analysis of mice in four different groups was carried out after intravenous tail injection. The data are displayed as the mean ± standard deviation (*n* = 5). Routine blood examination revealed various parameters, including white blood cell (WBC) count, red blood cell (RBC) count, hemoglobin (HGB) level, hematocrit (HCT), aspartate aminotransferase (AST) level, alanine aminotransferase (ALT) level, and platelet (PLT) count. (**B**) H&E histological staining of vital organs from both the control and treated groups, and no pathological changes were observed (scale bar, 100 μm)

Extensive evaluations were performed to determine the biosafety of NPs, including the analysis of blood samples and major organs from treated mice. H&E staining of the heart, liver, spleen, lungs, and kidneys displayed no significant morphological alterations or tissue damage in any of the groups (Fig. 8A), suggesting that the NPs did not induce any apparent organ toxicity. Further, a comprehensive blood analysis was carried out to examine potential inflammatory responses or organ dysfunction caused by NP treatments. The results, showcased in Fig. 8B, indicated that all key blood parameters remained within normal ranges. This includes markers for liver and kidney function, as well as other critical biochemical indicators. The alignment of these values within reference ranges reinforces the NPs’ compatibility and safety for biomedical applications. These findings collectively underscore the safety profile of Fe_x_Mo_y_S-PEG NPs, indicating they do not elicit harmful physiological responses in treated mice. This is crucial for advancing their use in clinical settings, particularly for cancer treatment strategies.

### Potential treatment of CRC patients with NPs

**Fig. 9:**
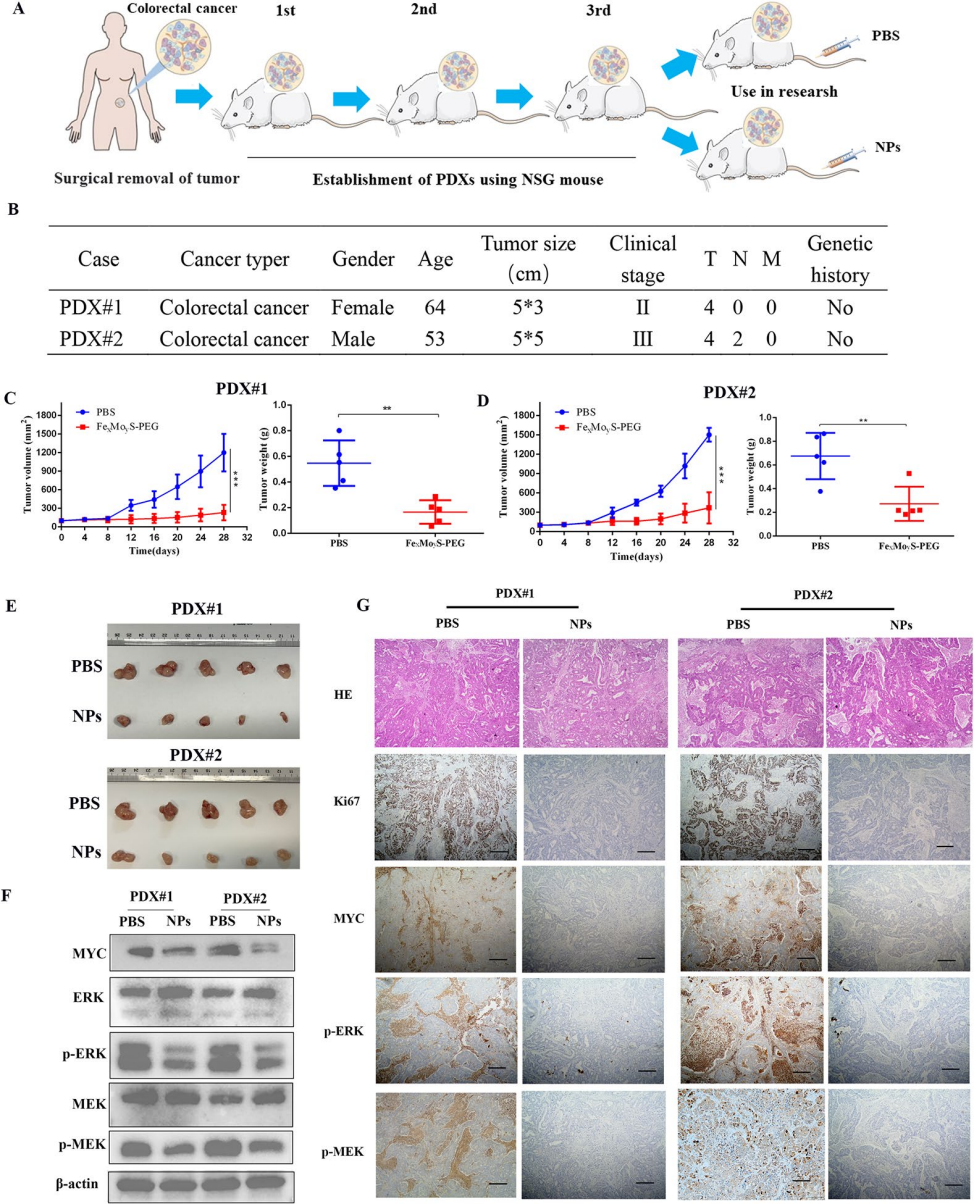
Fe_x_Mo_y_S-PEG NPs are potential treatments for CRC patients. (**A**) Construction of a PDX model of human CRC. (**B**) Clinical information for PDX#1 and 2 from patient cancer tissues. (**C**) and (**D**) Growth curves and tumor weights of PDX model tumors were evaluated following treatment with Fe_x_Mo_y_S-PEG (*n* = 5 per group). (**E**) Tumor photographs. (**F**) Western blot analysis of MYC, ERK, p-ERK, MEK and p-MEK expression in PDX tumors. β-actin served as a loading control. (**G**) H&E staining and Ki67, MYC, p-MEK, and p-ERK levels in harvested tissues were assessed via immunohistochemistry. Representative photographs of the different groups are shown (scale bar, 100 μm). ** *P* < 0.01, ***P < 0.001

The PDX model, established by transplanting human tumor specimens into immunodeficient mice, mirrors the histopathological features and growth patterns of the primary tumor while maintaining the heterogeneity of human cancer molecular characteristics^51^. This model serves as an alternative to human clinical trials for assessing the responsiveness to chemotherapy or NPs, making it valuable for personalized cancer treatment strategies. The creation of a CRC PDX model is illustrated in Fig. 9A. In this research, two specific PDX models, referred to as PDX#1 and PDX#2, were treated with NPs to evaluate their therapeutic efficacy. Clinical details pertaining to these models are summarized in Fig. 9B. The results indicated a significant reduction in tumor growth in the NPs group compared to the PBS group, with noticeable decreases in both tumor volume and weight (Fig. 9C and D). Further in vitro validation indicated that the NPs exerted tumor-suppressive effects by inhibiting the MAPK signaling pathway. IHC analysis demonstrated decreased expression of key proteins associated with this pathway, such as c-Myc, p-MEK1/2, and p-ERK1/2 in NP-treated tumor samples (Fig. 9E), aligning with the observed reductions in tumor size. Additionally, RNA and protein extractions from tumor tissues of different PDX groups facilitated further investigation into glycolysis-related gene expression. Western blot analysis revealed that the expression levels of p-MEK1/2 and p-ERK1/2 were significantly lower in the NPs group compared to the PBS group (Fig. 9F). Moreover, IHC analysis showed that the levels of Ki-67, MYC, p-MEK and p-ERK were lower in treatment group compared to control group, which were consistent with the tumor burdens in different groups (Fig. 9G). These results suggest that NPs can inhibit CRC tumor growth by affecting the MAPK pathway, which subsequently impacts glycolysis and tumor proliferation. Overall, these findings present strong evidence supporting the use of NPs as a viable approach to impede CRC tumor growth through modulation of the MAPK signaling pathway, impacting glycolysis and inhibiting tumor expansion in vivo.

## Conclusion

In this investigation, Fe_x_Mo_y_S-PEG NPs were synthesized via thermal decomposition and subsequently coated with PEG to enhance their stability and biocompatibility. These Fe_x_Mo_y_S-PEG NPs possess multivalent metal elements (Fe^2+/3+^ and Mo^4+/6+^), enabling efficient catalytic conversion of H_2_O_2_ into •OH, thus facilitating ROS-mediated dynamic therapy specifically targeting CRC cells. High-valence states of the metal ions (Fe^3+^/Mo^6+^) contribute to GSH depletion, enhancing oxidative stress within cancer cells and promoting a therapeutic response characterized by ferroptosis and suppressed glycolysis. Moreover, elevated ROS levels were found to inhibit the MAPK signaling pathway, affecting cell proliferation, apoptosis, metastasis, and metabolic activities. Our RNA sequencing analysis suggested that the impact of Fe_x_Mo_y_S-PEG NPs extended to metabolic and possibly immune functions within CRC cells, although the exploration of the NPs’ effect on tumor immunity remains incomplete due to resource constraints. In the in vivo environment, Fe_x_Mo_y_S-PEG NPs demonstrated significant tumor growth inhibition in mouse models carrying CRC and PDXs, underscoring their potential for clinical application. These comprehensive findings underscore the therapeutic potential of Fe_x_Mo_y_S-PEG NPs in treating CRC by altering the TME and metabolic pathways. The promising results in tumor inhibition and biological safety suggest that these NPs hold valuable prospects for future cancer treatment advancements. Further investigations will aim to elucidate the interaction between NPs and tumor immunity, enriching the therapeutic landscape of NP-mediated cancer treatment.

### Electronic supplementary material

Below is the link to the electronic supplementary material.

Supplementary Material 1

## Data Availability

All data generated or analyzed during this study are included in this published article.
