# Supplementary material for: Therapeutic potentials of FexMoyS-PEG nanoparticles in colorectal cancer: a multimodal approach via ROS-ferroptosis-glycolysis regulation
Source: J Nanobiotechnology. 2024 May 16;22:253. doi: 10.1186/s12951-024-02515-3 (PMC11097533; doi:10.1186/s12951-024-02515-3)
Supplement: Supplementary file 1 — Supplementary Material 1 [file 12951_2024_2515_MOESM1_ESM.docx]

Table S1. The sequences of primers for qRT-PCR

| Sequence (5’-3’) | |
| --- | --- |
| MYC forward | GTCTGGATCACCTTCTGCTGGAG |
| MYC reverse | GCTGCGTAGTTGTGCTGATGTG |
| GLUT1 forward | AGCCTCTCACTCTCCAGATACCTC |
| GLUT1 reverse | TACCACCTCACAGCAACTCTACAAG |
| LDHA forward | AGTGAGTAGGAGGCAGAGGTGAG |
| LDHA reverse | TGGGAGGGCATGGGATGAGG |
| GAPDH forward | GTCTCCTCTGACTTCAACAGCG |
| GAPDH reverse | ACCACCCTGTTGCTGTAGCCAA |





Figure S1 The hydrodynamic size of NPs.


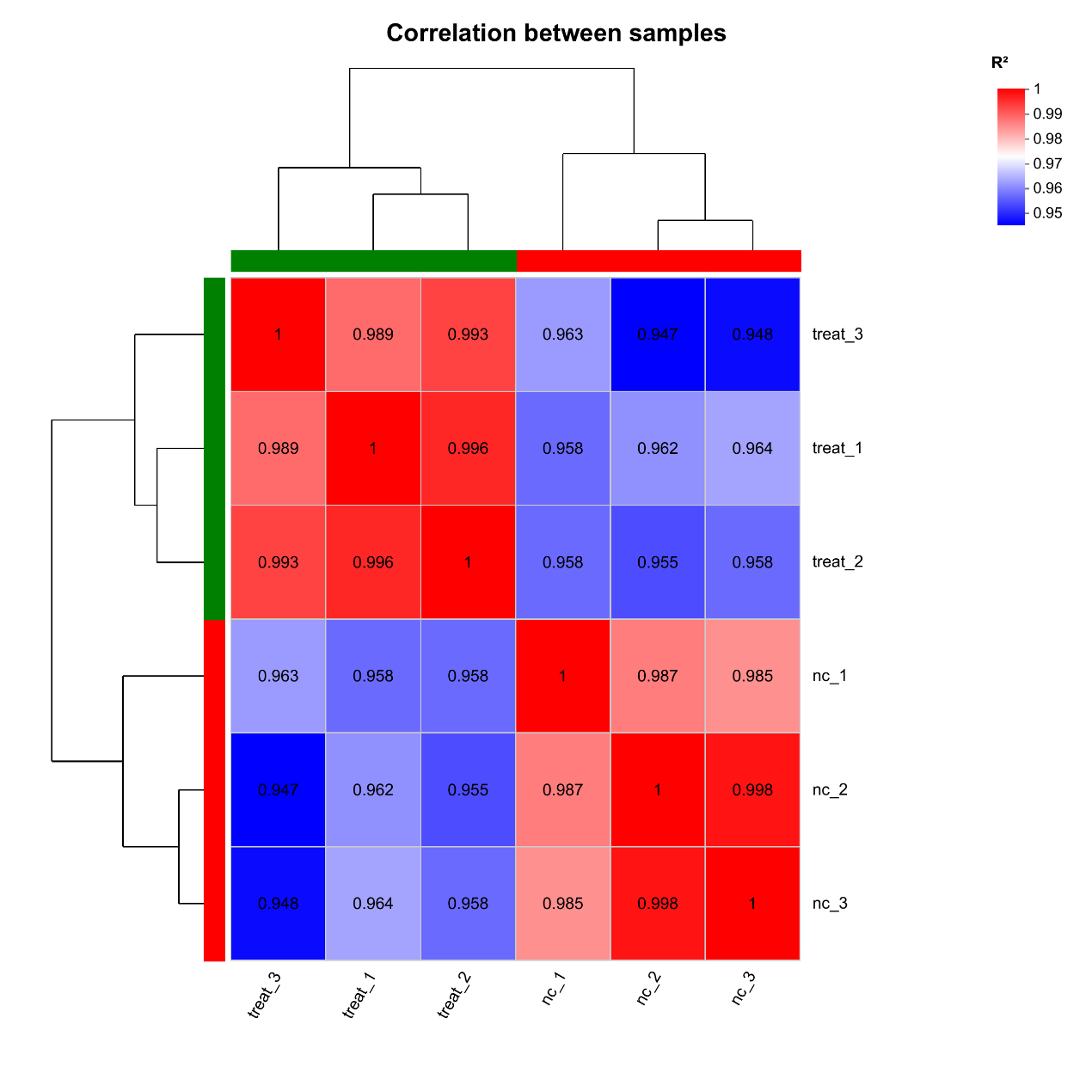


Figure S2. Unsupervised hierarchical clustering of the RNA-Seq data from the NC groups

and treat (NPs+ H_2_O_2_ )groups
